# Salmonella typhi Bacteremia With Infective Fasciitis and Psoas Abscess: A Rare and Challenging Presentation

**DOI:** 10.7759/cureus.85447

**Published:** 2025-06-06

**Authors:** Usamah Al-Anbagi, Abdulrahman Saad, Tarek Ibrahim, Hassan M Lameen, Abdulqadir J Nashwan

**Affiliations:** 1 Internal Medicine Department, Hazm Mebaireek General Hospital/Hamad Medical Corporation, Doha, QAT; 2 Medicine Department, Ministry of Public Health, Doha, QAT; 3 Pharmacy Department, Hamad Medical Corporation, Doha, QAT; 4 Radiology Department, Hamad Medical Corporation, Doha, QAT; 5 Nursing & Midwifery Research Department, Hamad Medical Corporation, Doha, QAT

**Keywords:** antibiotic, enteric fever, magnetic resonance imaging (mri), psoas abscess, salmonella typhi (s. typhi)

## Abstract

Enteric fever, caused by *Salmonella* *typhi* or *paratyphi*, remains a significant global health issue, especially in endemic areas. The disease typically presents with prolonged fever, abdominal discomfort, and gastrointestinal symptoms. However, complications such as infective fasciitis and muscle abscess, though rare, can occur and lead to severe consequences. We present a case of a 29-year-old male diagnosed with *S. typhi* infection, who developed infective fasciitis of the lower psoas and iliacus muscles, with early abscess formation. This case underscores the potential for atypical presentations of enteric fever and highlights the importance of early diagnosis and appropriate antibiotic therapy to prevent severe complications. This report also discusses diagnostic challenges, treatment options, and the evolving resistance patterns to common antibiotics to manage enteric fever.

## Introduction

Enteric fever, primarily caused by *Salmonella typhi* and *S. paratyphi*, remains a significant infectious disease in regions with inadequate sanitation and limited access to clean drinking water [1]. It is characterized by prolonged fever, gastrointestinal symptoms, and general malaise, often leading to misdiagnosis due to its nonspecific clinical presentation [1]. While complications of enteric fever are relatively rare, they can be severe and include conditions such as gastrointestinal perforation, hemorrhage, and, as seen in this case, infective fasciitis [2]. Infective fasciitis, especially involving deep muscle groups like the psoas and iliacus muscles, is an uncommon but potentially life-threatening complication of systemic infections such as enteric fever [2]. This case report highlights the unusual progression of *S. typhi* infection into infective fasciitis and psoas muscle abscess formation, illustrating the importance of recognizing atypical presentations of enteric fever and the critical need for early intervention and appropriate antimicrobial therapy. The increasing prevalence of antimicrobial resistance in endemic regions further complicates the management of this disease, necessitating careful selection of therapeutic agents based on local resistance patterns.

## Case presentation

A 29-year-old male with no known risk factors and who was immunocompetent presented with a three-day history of high-grade intermittent fever associated with sweating and localized right-sided hip and lower back pain, rated 6 out of 10 on the Numerical Rating Scale (NRS). The pain was non-radiating and confined to that region, with no involvement of other joints. He also reported a single episode of vomiting but denied chills, rigors, jaundice, skin rash, bleeding from any orifice, or changes in urine color or output. The patient denies any history of recent trauma, spinal or abdominal surgery, intravenous drug use, diabetes mellitus, or history of tuberculosis infection.

On examination, he was fully conscious, oriented, and appeared unwell. His vital signs showed a temperature of 39.9°C, a heart rate of 106 bpm (sinus rhythm), a respiratory rate of 19 breaths/minute, a blood pressure of 128/77 mmHg, and oxygen saturation of 99% on room air. He was mildly dehydrated but not jaundiced or pale, with no lymphadenopathy, lower limb edema, or signs of deep vein thrombosis (DVT). There was localized tenderness over the posterior right hip, particularly in the region of the sacroiliac joint and adjacent gluteal area. Cardiovascular and respiratory examinations were unremarkable. The abdomen was soft and non-tender, with no organomegaly, bowel sounds were present and normal, and the neurological exam was normal.

Initial investigations revealed a normal white blood cell count, markedly elevated C-reactive protein (CRP), and prolonged international normalized ratio (INR) and prothrombin time (PT). Other routine labs were within normal limits (Table 1). The chest and abdominal X-rays were unremarkable.

**Table 1:** Laboratory Investigations WBC: white blood cells (total leukocytes); HCT: hematocrit; Hb: hemoglobin; PLT: platelet count; Urea: serum urea; Cr: serum creatinine; K⁺: serum potassium; Na⁺: serum sodium; Ca²⁺: serum calcium; TP: total protein; Alb: albumin; ALT: alanine aminotransferase; AST: aspartate aminotransferase; ALP: alkaline phosphatase; TBil: total bilirubin; CRP: C-reactive protein; PCT: procalcitonin; PT: prothrombin time; INR: international normalized ratio; APTT: activated partial thromboplastin time; HIV: human immunodeficiency virus; HbA1c: hemoglobin A1c.

Parameters	On admission	Fourth day	On discharge	After 1 month (follow-up)	Reference values
Total leukocytes (x10^3^/uL)	7.9	7.6	9.2	6.8	6.2
Hematocrit (%)	43.9	34.9	35.6	44.1	40-50
Hemoglobin (g/dL)	15.3	11.9	11.9	14.6	13-17
Platelet (x10^3^/uL)	146	168	405	245	150-410
Serum urea (mmol/L)	3.7	3.5	4.3	2.6	2.5-7.8
Serum creatinine (umol/L)	101	57	65	75	62-106
Serum potassium K (mmol/L)	3.2	4	4	3.6	3.5-5.3
Serum sodium (mmol/L)	133	136	138	140	133-146
Serum calcium (mmol/L)	2.39	-	-	-	2.2-2.6
Serum total protein (g/L)	82	64	68	83	60-80
Serum albumin (g/L)	37	22	28	42	35-50
ALT (IU/L)	35	103	129	34	0-41
AST (IU/L)	38	110	48	24	0-41
Alkaline phosphatase (U/L)	63	72	72	70	40-129
Serum total bilirubin (mg/dL)	20	9	8	23	0-21
CRP (mg/L)	193	89	5.7	<2	0-5
Procalcitonin (ng/mL)	3.79	-	-	-	<0.05
PT (seconds)		13.9	12.4	-	9.4-12.5
INR	1.7	1.2	1.1	-	<1
APTT (seconds)	35	32	32	-	25.1-36.5
HIV	Negative				Negative
HbA1c (%)	4.7				4.0-5.6

The patient was admitted with a provisional diagnosis of infection of unknown source with suspicion of enteric fever, and started empirically on ceftriaxone 2 g IV daily along with paracetamol for symptomatic relief. On the third day, blood cultures grew *S. typhi*, sensitive to ceftriaxone, azithromycin, and ampicillin, and resistant to cefuroxime, so the antibiotic regimen was continued.

**Figure 1:**
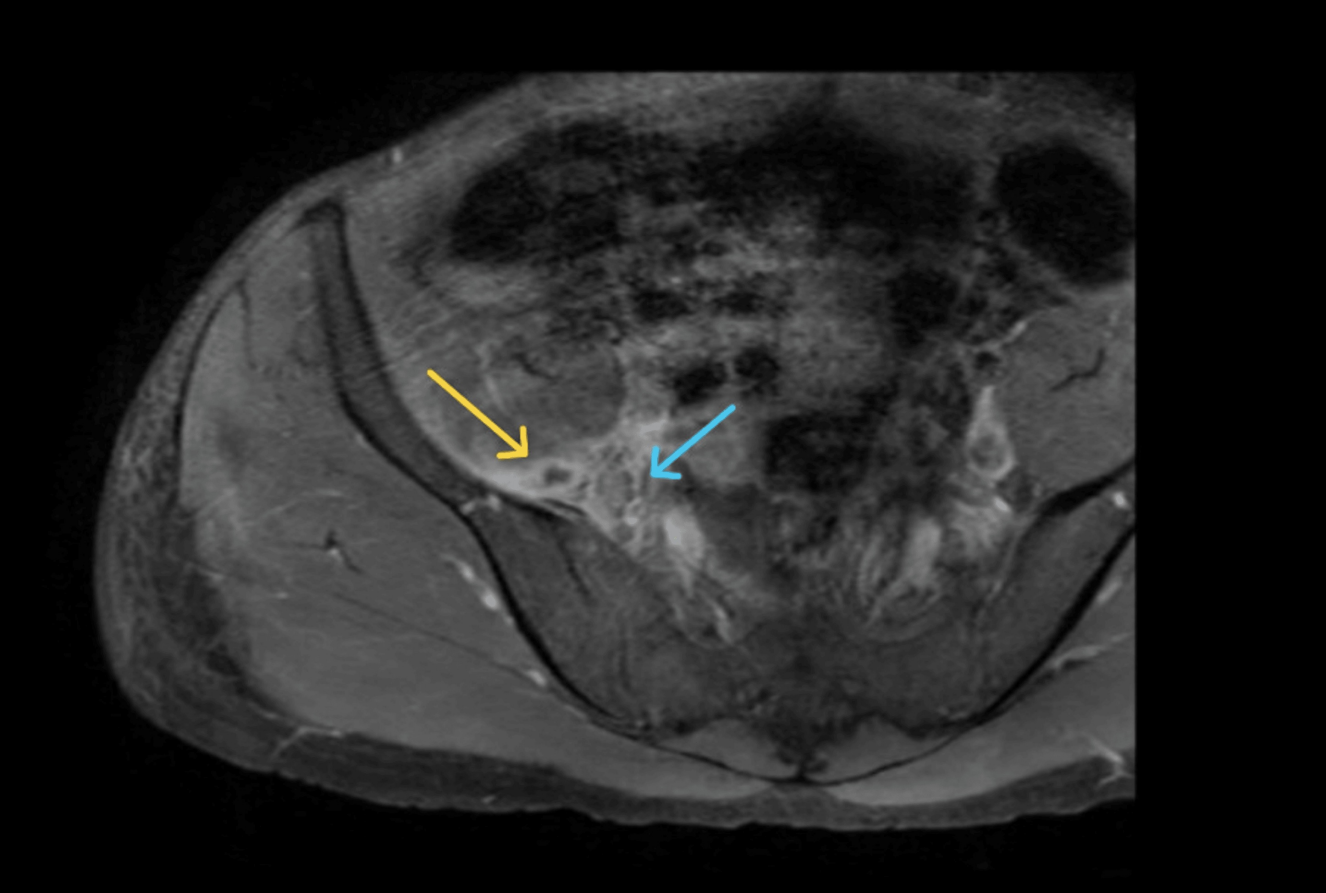
MRI T1-Weighted Image of the Pelvic Region MRI T1-weighted image reveals a small abscess collection (yellow arrow) and an anterior enhancing deep soft tissue adjacent to the para-sacroiliac joint, suggestive of fasciitis (blue arrow). MRI: magnetic resonance imaging.

The patient’s fever and general condition gradually improved, but he continued to experience severe right hip and lower abdominal pain with right hip restricted movement, requiring morphine for pain control. A hip X-ray was normal, while MRI of the hip and pelvis confirmed infective fasciitis involving the lower psoas and iliacus muscles, with small abscess formation (Figure 1).

**Figure 2:**
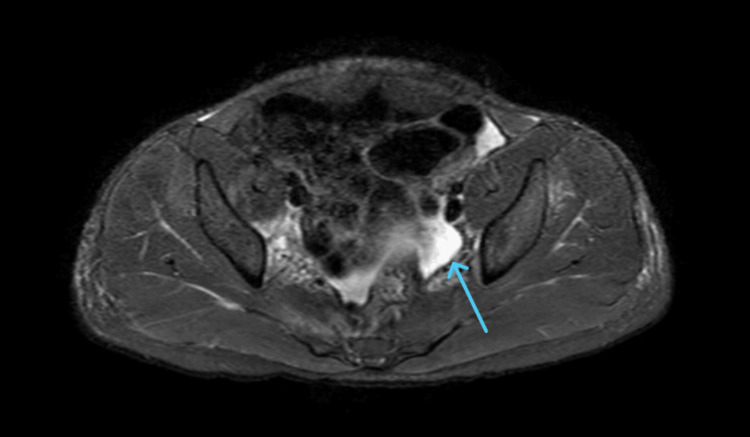
STIR Sequence of the Pelvis STIR sequence of the pelvis shows a small amount of free fluid in the pelvic cavity (blue arrow). STIR: short tau inversion recovery.

The pelvis's STIR (short tau inversion recovery) sequence also demonstrated a small amount of free fluid in the pelvic region (Figure 2). After a multidisciplinary discussion involving orthopedics, infectious disease, and interventional radiology teams, a decision was made to manage the abscess conservatively with antibiotics alone. The patient remained hospitalized for 12 days, and his condition improved significantly, although he continued to have some hip discomfort and movement limitation.

He was discharged on oral amoxicillin to complete six weeks of antibiotics. On follow-up at one month and again at two months, he reported significant improvement, with resolution of most of the pain and restoration of normal mobility. He was able to ambulate independently with no restriction in movement.

## Discussion

A 29-year-old male with no significant medical history presented with a three-day history of fever, abdominal pain, and diarrhea. Initial clinical examination suggested enteric fever, and blood cultures confirmed the presence of *S. typhi*. His clinical course was complicated by the development of infective fasciitis in the lower psoas and iliacus muscles, with abscess formation rare but serious complication of enteric fever. Fortunately, he responded well to prompt antibiotic therapy without requiring surgical or percutaneous intervention. This case highlights the importance of considering unusual complications of enteric fever, especially in regions with prevalent antimicrobial resistance. The patient's favorable response to third-generation cephalosporins underscores the need for careful antibiotic selection based on local resistance patterns.

*Salmonella enterica* serotype *typhi* (*S. typhi*) is the primary organism responsible for enteric (typhoid) fever. However, other serotypes such as Paratyphi A, B, and C can produce a similar clinical syndrome [1]. The highest incidence of *S. typhi* infection is observed in regions like South-Central Asia, Southeast Asia [2], and parts of Southern Africa, with urban areas showing significantly higher rates ranging from 576 to 1173 cases per 100,000 child-years compared to rural settings [3]. Risk factors include poor sanitation, larger household size, and limited access to clean water or hygienic facilities. Transmission dynamics may differ between serotypes: while typhoid fever often spreads within households (e.g., via shared utensils or inadequate toilet facilities), paratyphoid fever is more commonly associated with foodborne exposure, such as street food [4]. Notably, *S. paratyphi* is an emerging concern among vaccinated travelers, as current typhoid vaccines offer limited protection against *S. paratyphi*, which lacks this antigen [5].

Enteric fever is a systemic illness, and its symptoms typically begin 5-21 days after exposure and include fever, abdominal pain, and gastrointestinal disturbances such as diarrhea or constipation. Classic signs like stepwise fever, rose spots, and hepatosplenomegaly are now less common due to early antibiotic use [6]. Serious complications, including intestinal perforation and septic shock, may occur in advanced cases, especially where treatment is delayed. The clinical presentation can vary based on age, geographic region, and access to care [7]. Neurological manifestations of enteric fever include headache and, in severe cases, typhoid encephalopathy, characterized by delirium, confusion, and altered consciousness [8].

Regarding musculoskeletal involvement, enteric fever can lead to arthralgias and myalgias in about 20% of patients [9]. In some cases, bacteremic spread may result in focal musculoskeletal infections, although these are rare. While specific data on enteric fever-related myositis or septic arthritis are limited, musculoskeletal symptoms are significant and can complicate the disease [10].

Psoas abscess, a collection of pus in the iliopsoas muscle compartment, may occur as either psoas pyomyositis (a purulent infection of the iliopsoas muscle) or with an associated abscess [11]. Primary psoas infections typically result from hematogenous seeding, often in individuals with risk factors like diabetes, intravenous drug use, renal failure, or human immunodeficiency virus (HIV) [12]. Secondary infections occur due to direct spread from neighboring tissues [12]. The clinical presentation is nonspecific, often involving fever, back pain, and limp, but this classic "triad" (fever, back pain, and limp) appears in only 30% of cases [13]. The infection progresses in stages: Stage 1 involves insidious onset of flank or low back pain (early pyomyositis), Stage 2 is marked by more localized pain as an abscess forms, and Stage 3 presents with systemic toxic symptoms such as high fever or septic shock [13]. Psoas infections may also lead to complications like septic shock, deep venous thrombosis, hydronephrosis, bowel ileus, and hip septic arthritis.

Enteric fever should be considered in febrile patients living in or traveling from endemic areas, especially if they have gastrointestinal symptoms like abdominal pain, diarrhea, or constipation. Blood and stool cultures are essential for diagnosis, though blood culture yields are not highly sensitive (50-70%), and results take several days to obtain [14]. Stool culture may be positive in 30-40% of cases, but it is often negative when patients seek medical care [15]. Polymerase chain reaction (PCR) tests have limited sensitivity due to the low bacterial concentration in blood [16]. The Widal test detects antibodies against *S. typhi* but is of limited use in endemic areas, as it may indicate past infection [17]. New tests targeting serum IgA against hemolysin E are promising. ELISA (enzyme-linked immunosorbent assay) for anti-Vi antibodies helps detect carriers but is not useful for diagnosing acute infections due to high background levels in endemic areas [18].

Enteric fever is primarily treated with a single antibacterial drug, selected based on illness severity, local resistance patterns, and available resources. Common choices include fluoroquinolones, third-generation cephalosporins, and azithromycin, with carbapenems reserved for extensively drug-resistant strains. Severe cases typically require parenteral therapy, with ceftriaxone or meropenem used depending on the geographic region of infection, while azithromycin is often used in areas with fluoroquinolone resistance [19]. Antibiotic therapy is adjusted based on susceptibility testing, and adjunctive corticosteroids may be considered for severe infections. Fluoroquinolone resistance, particularly in South Asia, has shifted treatment protocols, with azithromycin and third-generation cephalosporins becoming more widely used [20].

## Conclusions

This case emphasizes the need for heightened clinical awareness regarding the potential for unusual complications, such as infective fasciitis and muscle abscess, in patients with enteric fever. Early identification and appropriate antibiotic therapy are crucial for managing such complications. Given the evolving antimicrobial resistance, especially in endemic regions, careful antibiotic selection guided by local resistance patterns is vital for successful outcomes. This report serves as a reminder of the broad differential diagnosis for fever in endemic regions and the necessity for timely, effective interventions to prevent morbidity and mortality from enteric fever-related complications.
